# Supplementary material for: IMAGE-IN: Interactive web-based multidimensional 3D visualizer for multi-modal microscopy images
Source: PLoS One. 2022 Dec 30;17(12):e0279825. doi: 10.1371/journal.pone.0279825 (PMC9803232; doi:10.1371/journal.pone.0279825)
Supplement: Attachment — Submitted filename: Response to Reviewers.pdf [file pone.0279825.s001.pdf]

Dear editor, dear reviewers,

Thank you for reviewing and providing helpful comments on our manuscript, "**IMAGE-IN: Interactive Web-based Multidimensional 3D Visualizer for Multi-Modal Microscopy Images**" submitted to PLoS One. Please find a detailed reply to each comment below.

Kind regards,
Yubraj Gupta

Editor/Journal Requirements:

-- Thank you for your comments. We wrote an article using the PLOS ONE latex template. With respect to code sharing, we have provided GitHub links as well as a DOI link in the reference section: (https://doi.org/10.5281/zenodo.7351555). In the editorial manager, the corresponding author registered his ORCID ID. We double-checked the reference section and found no errors. We added one extra reference to our manuscript since we obtained one image from the Sparc database for our study and so needed to cite the data provider. And lastly, we have processed all figures using the Preflight Analysis and Conversion Engine (PACE) digital diagnostic tool to adhere to the PLOS One standard image in .tif files.

"Yuan PQ, Wang L, Mulugeta M, Tache Y. CLARITY and three-dimensional (3D) imaging of the mouse and porcine colonic innervation; 2022. Available from: https://sparc.science/datasets/31/version/4."

Reviewer 1:

- In the methodology for IMAGE_IN comparison, I hoped to use a commonly used close source 3D rendering application beside ImageJ, the open one.

-- Thanks for the suggestion to compare both to open-source and commercial software. We therefore now compare the presented IMAGE-IN web viewer to ClearVolume (the ImageJ open-source desktop viewer), Napari (the Python desktop viewer), and Imaris (the closed-source desktop viewer).

We changed the text (line numbers 425–449) and Figures 8 and 9 show the results:

"Comparison between IMAGE-IN, ClearVolume, Napari, and Imaris viewers"

"In this section, we compared the results acquired from IMAGE-IN to the publicly available ClearVolume (a renderer running inside ImageJ) and Napari (a renderer running inside Python) viewers, as well as with the commercial Imaris (a closed-source desktop viewer) viewer. Figs 8 and 9 show the comparative results of each visualizer when two-channel submandibular ganglion [45] and three-channel mouse proximal colon [44] CLSM microscope images were passed through them. Aside from modifying mouse manipulators such as zoom and panning, these image screenshots were captured without adjusting any parameters. IMAGE-IN, ClearVolume, and Imaris viewers provide functionality such as region of interest clipping, voxel dimension scaling, an opacity slider, a color picker, and more. However, the native Napari Python visualizer does not provide region of interest clipping or voxel dimension scalings. ClearVolume only supports maximum intensity projection to render volumetric data; however, our visualizer offers three different blending modes to render volumetric data: composite (default), maximum, and minimum intensity projection. Napari offers five blending modes: additive, average, maximum (selected by default), and minimum intensity projection. Imaris, for example, offers four blending modes: MIP (maximum intensity projection), normal shading, Blend, and shadow projection. To render the provided input CLSM data, we use the maximum intensity projection mode in all three visualizers (Figs 8 and 9).

Our visualizer provides surface (3D isosurface), tri-planar (cross-sections), and MPR (orthogonal sections) viewers. The rendered image quality is nearly identical in all viewers. The user interface (UI) in IMAGE-IN is substantially quicker than in Napari and ClearVolume viewer. However, the commercial (closed source) Imaris viewer is faster than all three viewers."

- I did not get why in the results they have focused on IMAGE_IN performance on laptop and desktop comparison on loading and render time. Maybe if the comparison was about IMAGE_IN performance between laptop and mobile systems.

-- The IMAGE-IN web viewer's rendering speed and image quality heavily rely on the client's (frontend side) computer power. This is due to the IMAGE-IN design, in which the backend is solely needed to run the Django web framework (server) in order to host the webpage; the backend has no impact on loading and rendering the data, which is entirely handled on the frontend. To give a realistic idea about the rendering limit on a typical desktop and laptop, we, therefore, measured and presented their rendering performance.

Thanks for suggesting to also compare to mobile devices. We now also present IMAGE-IN rendering times on an iPad Air (4th generation) in Table 3.

Changed text here: line numbers 535–564.

"In addition, we tested the IMAGE-IN web-viewer performance on the iPad Air (3rd generation, 64 Gb). Because the computational graphical power of the iPad is limited, we pass three small data sets (three-channel mouse proximal colon-8 (147.7 Mb) [44], two-channel submandibular ganglion-9 (68.2 Mb) [45], and two-channel mouse proximal colon-10 (297.8 Mb) [46]) to measure the loading and rendering time performance on the iPad device, and later we pass the same three data sets from laptop and desktop for comparison purposes, as shown in Table 2. Table 3 shows the time taken by the iPad device to load and render the respective (three-channel mouse proximal colon-8 (147.7Mb) [44], two-channel submandibular ganglion-9 (68.2 Mb) [45], and two-channel mouse proximal colon-10 (297.8 Mb) [46]) image data in a Google Chrome web browser. For the three-channel mouse proximal colon-8 [44] data, the iPad device took 2273 and 7069 ms to render it into volume and surface, as shown in Table 3, which are high compared to laptop and desktop performance for the same input (as shown in Table 2). Moreover, in the case of two-channel submandibular ganglion-9 (68.2 Mb) [45], a similar pattern can be noticed; the iPad device requires more computing power to render them into volume and surface than the other two devices. Furthermore, in the case of volume rendering for the two-channel mouse proximal colon-10 (297.8 Mb) [46], iPad device took 2788 ms, which is again excessive when compared to laptop (2086.6 ms) and desktop (2275.75 ms) devices. When the same two-channel mouse proximal colon-10 (297.8 Mb) [46] data was surface rendered, the iPad device web-browser began to crash. This is because the surface rendering algorithm demands a lot of computing power to compute marching cubes, and this also happens with larger data sets. However, the same two-channel mouse proximal colon-10 (297.8 Mb) [46] successfully renders onto the surface in the case of laptop and desktop devices, and their loading and rendering are shown in Table 2.

In the case of the iPad device, the rendered image quality and interaction smoothness were satisfactory in all viewers for all small datasets, with the exception of a slight lag in the case of volume rendering in composite blend mode for the two-channel mouse proximal colon-10 (297.8 Mb); nevertheless, for large datasets, such as those > 300 Mb, we observed a web-browser crash in the case of surface and volume rendering."

- I found the tri-planner and the multi-planner viewer that are provided with this system is of great privilege of IMAGE-IN when compared for example to ImageJ application, yet I did not see that the authors have highlighted the advantages of having them.

-- Thank you for stressing this unique selling point. We liked the idea and therefore now highlighted the features of the tri-planar and MPR viewers in line numbers 612–621.

Changed text here: line numbers 612-621

“Similarly, in the case of the tri-planar viewer, we have included three cross-sectional views, namely, XY, XZ, and YZ axes, and each axis has visibility on/off features, which allows microbiologists to perform image analysis based on their preferred axis, as shown in Fig 13 where we have rendered a two-channel mouse proximal colon [46] in a tri-planar viewer. This viewer also has interactive mouse selector features such as zooming, rotation, and panning. Likewise, in the case of the MPR web viewer, we have implemented three orthogonal plane viewers, namely, axial, coronal, and sagittal, to aid microbiologists in exploring insights while undertaking deep analysis of the volume data. Both tri-planar and MPR viewers provide window and colour level increase and decrease features that help to maintain the contrast and brightness of the rendered data.”

- Comparison between IMAGE-IN, ClearVolume, and Napari viewer, is the best part in results and discussion section maybe moving it to the front of the topic is better.

-- We agree to this good suggestion and have moved the comparison section from line numbers 544-567 further to the front (425-449).

- What I found not handy while using IMAGE-IN is the necessity of converting the images into DICOM format. I believe that IMAGE-IN web will be of great value if it is more flexible regarding data format like lif rather than accepting DICOM only.

-- This was a valid drawback of our viewer, which is why we now invested the extra effort (Bioformats is not yet available in JavaScript) to support more file formats. The new edition of the IMAGE-IN web viewer now supports .dcm, .tif, .tiff, .nrrd, .mha, .nii, .ome.tif, .png, .bmp, .jpg, and some other formats; however, due to the unavailability of a generic import tool in JavaScript, the IMAGE-IN viewer is still unable to import proprietary file formats such as .czi, .lif, .nd2, and others. Line numbers: 263-267.

“Our IMAGE-IN web-viewer can currently render input images belonging to .dcm, .tif, .tiff, .nrrd, .mha, .nii, .ome.tif, .png, .bmp, .jpg, and other formats; however, due to the unavailability of a generic import tool in JavaScript, the IMAGE-IN web-viewer is still unable to load proprietary files such as .czi, .lif, .nd2.”

Reviewer 2:

- The spelling of the short title must be corrected “IMAGE-IN multidimensisional web viewer”.

-- Thanks for picking up this mistake, which we now corrected to "IMAGE-IN multidimensional web viewer".

- Lines 98,99,100 are repetition.

-- Thanks for picking this up. We now have deleted the below paragraph from the manuscript (99-103).

“Furthermore, the purpose of this study (task-2) is to provide an interactive web-based multidimensional 3D visualizer for users that allows them to visualize any dicomized multidimensional image in a web browser rather than on a local computer where an installer file of the targeted OS visualizer of a specific vendor must be installed.”

- Table 2 presents the performance of the software using laptop and desktop, the results were presented in msec, how did you eliminate the effect of internet services? It would be really helpful if the comparison was after a time interval rather than laptop and desktop so you can measure the reliability of the software.

-- Thanks for picking up on a potential misunderstanding about including the less reliable internet services in our time measurements. IMAGE-IN web viewer performance has been measured after the webpage is fully loaded on the client side, which means the internet service has no impact on the measurement. And furthermore, we are now measuring loading and image rendering times on three devices: a laptop, a desktop, and an iPad Air, to get an indication of how computing power affects the end-user experience. Consequently, we are now clarifying this by reporting both “Loading Time” and “Render Time” and changing the text to (453–468):

“Later, we evaluated the performance of the IMAGE-IN web viewer on the frontend rather than the backend because the backend is only used to host the Django web server and all other libraries (such as ITK.js and VTK.js) are loaded on the frontend when the user loads the webpage. As a result, we assess the rendering performance of the IMAGE-IN web viewer on the client side to determine the impact of the client’s computer graphical power on performance. Because of this, we chose three different devices, the first of which is a laptop with a system specification (Memory: 16 Gb, processor: Intel Core i7-10750H CPU 2.60Hz, Graphics: NVIDIA GeForce RTX 2060, OS type: 64-bit, max 3D Texture size: 16384), the second of which is a desktop with a system specification (Memory: 64 Gb, processor: Intel Core i7-6700K CPU 4Hz, Graphics: NVIDIA GeForce GTX TITAN, OS type: 64-bit, max 3D Texture size: 2048), both of which were running the Ubuntu 20.04.4 LTS (Focal Fossa) operating system and both of which had a Google Chrome and Firefox browser pre-installed, and the third device is an iPad Air (3rd generation, 64 Gb), to gain a better sense of how graphical computational power will affect the end-user experience.”

-- Likewise, we also measured the viewer performance on iPad Air, which can be seen in Table 3. Line numbers 535-564.

“In addition, we tested the IMAGE-IN web-viewer performance on the iPad Air (3rd generation, 64 Gb). Because the computational graphical power of the iPad is limited, we pass three small data sets (three-channel mouse proximal colon-8 (147.7 Mb) [44], two-channel submandibular ganglion-9 (68.2 Mb) [45], and two-channel mouse proximal colon-10 (297.8 Mb) [46]) to measure the loading and rendering time performance on the iPad device, and later we pass the same three data sets from laptop and desktop for comparison purposes, as shown in Table 2. Table 3 shows the time taken by the iPad device to load and render the respective (three-channel mouse proximal colon-8 (147.7Mb) [44], two-channel submandibular ganglion-9 (68.2 Mb) [45], and two-channel mouse proximal colon-10 (297.8 Mb) [46]) image data in a Google Chrome web browser. For the three-channel mouse proximal colon-8 [44] data, the iPad device took 2273 and 7069 ms to render it into volume and surface, as shown in Table 3, which are high compared to laptop and desktop performance for the same input (as shown in Table 2). Moreover, in the case of two-channel submandibular ganglion-9 (68.2 Mb) [45], a similar pattern can be noticed; the iPad device requires more computing power to render them into volume and surface than the other two devices. Furthermore, in the case of volume rendering for the two-channel mouse proximal colon-10 (297.8 Mb) [46], iPad device took 2788 ms, which is again excessive when compared to laptop (2086.6 ms) and desktop (2275.75 ms) devices. When the same two-channel mouse proximal colon-10 (297.8 Mb) [46] data was surface rendered, the iPad device web-browser began to crash. This is because the surface rendering algorithm demands a lot of computing power to compute marching cubes, and this also happens with larger data sets. However, the same two-channel mouse proximal colon-10 (297.8 Mb) [46] successfully renders onto the surface in the case of laptop and desktop devices, and their loading and rendering are shown in Table 2.

In the case of the iPad device, the rendered image quality and interaction smoothness were satisfactory in all viewers for all small datasets, with the exception of a slight lag in the case of volume rendering in composite blend mode for the two-channel mouse proximal colon-10 (297.8 Mb); nevertheless, for large datasets, such as those > 300 Mb, we observed a web-browser crash in the case of surface and volume rendering.”

Thank you for your comments.
